# DNA-Binding with One Finger (Dof) Transcription Factor Gene Family Study Reveals Differential Stress-Responsive Transcription Factors in Contrasting Drought Tolerance Potato Species

**DOI:** 10.3390/ijms25063488

**Published:** 2024-03-20

**Authors:** Xin Jin, Zemin Wang, Qianyi Ai, Xuan Li, Jiangwei Yang, Ning Zhang, Huaijun Si

**Affiliations:** 1 State Key Laboratory of Aridland Crop Science, Gansu Agricultural University, Lanzhou 730070, China; jinx@gsau.edu.cn (X.J.); wzm406433254@126.com (Z.W.); yyangjiangwei@126.com (J.Y.); ningzh@gsau.edu.cn (N.Z.); 2 College of Life Science and Technology, Gansu Agricultural University, Lanzhou 730070, China; 15163890231@163.com (Q.A.); 17794431180@163.com (X.L.)

**Keywords:** potato, Dof family, drought, stress response, CDF, lncRNA

## Abstract

DNA-binding with one finger (Dof) proteins comprise a large family that play central roles in stress tolerance by regulating the expression of stress-responsive genes via the DOFCORE element or by interacting with other regulatory proteins. Although the Dof TF has been identified in a variety of species, its systemic analysis in potato (*Solanum tuberosum* L.) is lacking and its potential role in abiotic stress responses remains unclear. A total of 36 potential Dof genes in potato were examined at the genomic and transcriptomic levels in this work. Five phylogenetic groups can be formed from these 36 Dof proteins. An analysis of cis-acting elements revealed the potential roles of Dofs in potato development, including under numerous abiotic stress conditions. The cycling Dof factors (CDFs) might be the initial step in the abiotic stress response signaling cascade. In potato, five CDFs (StCDF1/StDof19, StCDF2/StDof4, StCDF3/StDof11, StCDF4/StDof24, and StCDF5/StDof15) were identified, which are homologs of Arabidopsis CDFs. The results revealed that these genes were engaged in a variety of abiotic reactions. Moreover, an expression analysis of *StDof* genes in two potato cultivars (‘Long10′ (drought tolerant) and ‘DXY’ (drought susceptible)) of contrasting tolerances under drought stress was carried out. Further, a regulatory network mediated by lncRNA and its target Dofs was established. The present study provides fundamental knowledge for further investigation of the roles of Dofs in the adaptation of potato to drought stress, aiming to provide insights into a viable strategy for crop improvement and stress-resistance breeding.

## 1. Introduction

Transcription factors (TFs) are essential components of many regulatory and signaling networks in plants [1,2], which have been proven to be pivotal in plants’ responses to abiotic stress, including drought, and low and high temperatures. The survival, growth, and reproduction of plants are impacted by adverse climatic factors such as drought and extreme temperatures. Under the control of a vast array of stress-responsive genes and a sophisticated network of TFs, plants have evolved a variety of biochemical and physiological strategies to maintain growth and cell integrity in adverse environmental conditions. DNA-binding with one finger (Dof) proteins are a group of plant-specific TFs [3]. Following the isolation of the first *Dof* gene (*Zmdof1*) from maize, additional *Dof* genes have been identified in other plants and are the subject of extensive research [4,5,6,7,8]. In N-terminal regions, the Dof proteins contain a highly conserved Dof domain (about 52 amino acids with a Cys2/Cys2 zinc finger structure). T/AAAAG is a common core sequence of the Dof domain recognized in the promoters of target genes. Dof proteins also contain a C-terminal with a transcriptional activation function. Interestingly, the Dof domain can mediate not only protein–DNA interactions, but also protein–protein interactions [9]; for instance, Dof-OBPl (for OBF-binding protein) [10], Dof-bZIP [11], and Dof-MYB [12] have been characterized. At the same time, the conserved motif at the C-terminal of some Dof proteins also plays an important role in specific protein–protein interactions. For example, cycling Dof factor (CDF) proteins often play a regulatory role by interacting with post-translational regulatory proteins (such as the clock gene GIGANTEA (GI) and flavin binding kelch repeat F-box protein 1 (FKF1)) via a special motif in Dof proteins [6,13,14]. In addition, small ubiquitin-like modifier (SUMO) targets ubiquitin ligase (AT-STUbL4) and induces ubiquitination degradation of CDF2 by binding to it, thereby increasing CO mRNA levels and promoting flowering during the photoperiod [15]. These findings indicate that Dof TFs play a regulatory role in multiple dimensions by interacting with TFs and other proteins.

It has been demonstrated that Dofs are involved in the control of gene expression in a variety of physiological processes, including seed germination [16], flowering [17,18], root growth [19], light-mediated regulation [20], and hormone responses [21]. Dof TFs also play essential roles in the reaction to abiotic and biotic stresses, including drought [22,23], salt [24,25], extreme temperature [26,27], and pathogens [28]. The rose genome contains a total of 24 *Dof* genes, the majority of which have shown elevated expression levels in response to salt (in 21 cases) and drought (19 cases) stress conditions, highlighting the critical role of *Dof* genes in abiotic stress tolerance [29]. In walnut, JrDof3 directly regulates JrGRAS2, which regulates the expression of *HSPs* to improve heat resistance [30]. Wang et al. [8] isolated 11 *VaDof* genes (from a total of 25), which responded to cold stress in grape plants, and *VaDof17d*, which enhanced the cold tolerance of grape callus by increasing raffinose synthesis. Moreover, the C-repeat Binding Factor/Dehydration-Responsive Element (DRE) Binding Factor (CBF/DREB) transcriptional regulatory network plays a key role in plant abiotic stress. Recent results indicate that CDF plays a role in developmental processes and stress responses by participating in the GI pathway to regulate CBF regulators [31,32,33,34]. In Arabidopsis, *AtCDF3* overexpression enhanced the abiotic stress tolerance of transgenic plants and promoted late flowering by direct control of the GI-CDF module through regulating CBFs, DREB2A, and others, also including the GI-independent pathway [32,33]. The Arabidopsis CDF3 T-DNA insertion mutant *cdf3-1* was much more sensitive to drought and low temperature stress, whereas *CDF3* overexpression enhanced the tolerance to various abiotic stresses (drought, cold, and osmotic stress) of transgenic plants by regulating the CBF/DREB and ZAT10/12 pathways [28]. Dof proteins also play important roles in crop senescence, C/N regulation, photosynthesis regulation, and chloroplast development [6,16,35,36]. Some Dof TFs regulate both abiotic stress resistance and important agronomic traits in crops, such as flowering time and abiotic stress (TDDF1 and SlCDF3) in tomato [32,33,37], biomass and abiotic stress in tomato [33], and GhDof1 improved salt and cold tolerance and seed oil content in *Gossypium hirsutum* [38]. It is extremely important to harmonize and unify crop yield increases with the development of stress resistance and agronomic improvement.

Potato (*Solanum tuberosum* L.) is an important non-grain food crop worldwide, which is commonly sensitive to abiotic stress (drought, salinity, and heat, etc.). With increasing global climate change, there are further requirements for the study of potato abiotic resistance, which is a limiting factor affecting potato yield and quality. Finding and researching new abiotic resistance genes is necessary for a better understanding of the control mechanisms of resistance, as well as for future abiotic resistance breeding in potatoes. Despite the importance of Dofs in plant abiotic stress resistance, little is known about the function of Dofs in potato abiotic stress responses. StCDF1, a member of potato Dof TF, acted as a mediator between the circadian clock and the StSP6A mobile tuberization signal to regulate tuberization and potato life cycle length [14]. Recently, a study demonstrated that StCDF1, together with a long non-coding RNA (lncRNA) counterpart named *StFLORE*, also regulates water loss by affecting stomatal development and diurnal opening [14,39]. This study includes genome-wide identification of the putative *StDof* gene family in potato, the construction of a phylogenetic tree, and the annotation of the chromosomal position of these genes. We assessed the transcript abundance of the *StDof* genes in various abiotic conditions using whole-transcriptome data and qRT-PCR. Additionally, the expression profiles of specific *Dof* genes under drought stress were examined. The structure, chromosomal locations, and evolutionary relationships of the *Dof* gene family in the potato genome were also carefully examined. The expression profiles of the *StDofs* in various tissues and stress settings were analyzed to determine whether stress affected the expression levels of the *Dof* genes in potato. Moreover, a regulatory network mediated by lncRNA and its target *Dofs* was established. The findings offer baseline knowledge of an important gene family in potato species, which will form the basis for further functional analyses and for breeding stress-tolerant potato.

## 2. Results

### 2.1. Identification and Phylogenetic Analysis of Dof Genes in Potato

A total of 36 *Dof* genes were identified in *S. tuberosum* by combining HMM (e-value < 0.01) scan, BLAST (e-value < 1 × 10^−5^ search), and CDD search method. Except for *StDof1* and *StDof27*, the rest of the *Dof* genes’ accession numbers were obtained from GenBank. The 36 *Dof* genes in potato showed a variance in gene length, ranging from 498 bp (*StDof5*) to 1512 bp (*StDof15*) (Table 1).

**Figure 1:**
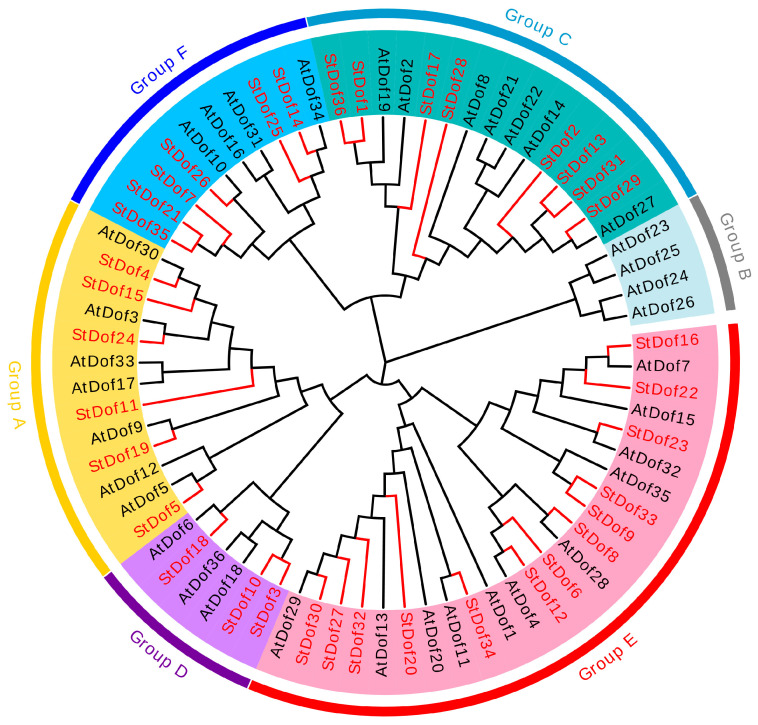
Phylogenetic analysis of the Dof genes in potato and Arabidopsis. The unrooted tree was generated by ClustalW in MEGA7 using the conserved amino acid sequences of the 36 StDof and 36 AtDof proteins. Dof proteins groups are distinguished by different colors.

Based on the conserved domain, the StDofs can be divided into five groups (Groups A–F, see Figure 1), with Group E being the largest group (containing 13 members) and Group D being the smallest (having only three members). However, Group B was not found in *S. tuberosum*. These outcomes align with those found in the grapevine.

### 2.2. Gene Structure, Chromosome Localization, and Duplication Analysis of StDof Genes

**Figure 2:**
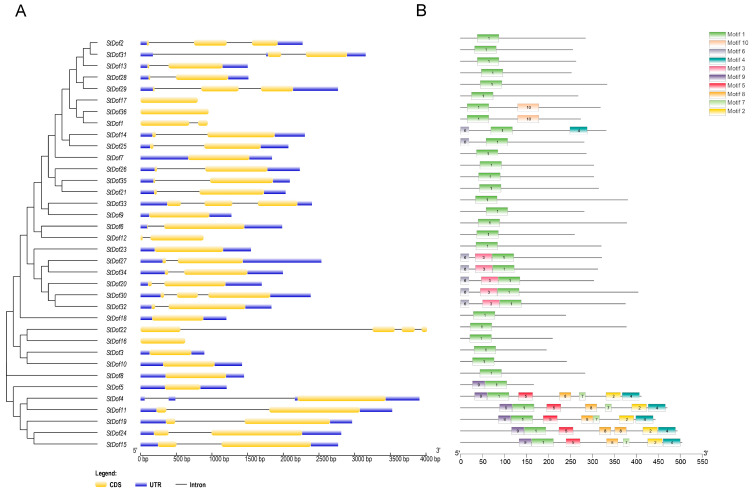
Characterization of potato *Dof* genes. (**A**) Potato *Dof* gene exon/intron structure. Exons are represented as yellow boxes. Introns are shown by black lines, whereas untranslated regions (UTRs) are represented by blue boxes. The size of the exons and introns can be estimated using the scale at the bottom. (**B**) Conserved motif distribution in the 36 StDof proteins. A number in a colored box represents each motif. For more details on the motifs, see Supplemental Table S1. Different motifs are represented by different colored double-sided wedge boxes.

The exon–intron organization was previously known to play critical roles in the evolution of several gene families. GSDS 2.0 (http://gsds.gao-lab.org/, accessed on 5 September 2022) was used to generate the exon–intron structure of 36 *StDof* genes. The gene structure (exon–intron) analysis revealed that *StDofs* had a maximum of four exons (Figure 2A). Members of Groups A and F had two exons and one intron (except *StDof4*, five genes in Group A and *StDof7* in Group F, which only had one exon), whereas members of Group C had three exons and two introns (Figure 2A). In Group E, there were four *StDof* genes containing one exon, six *StDof* genes containing two exons, two genes containing three exons (*StDof30* and *StDof33*), and only one *StDof* gene containing four exons. Furthermore, only one exon existed in all the members of Group D. Group E member *StDof22* contained four exons (Figure 2A). These results indicated that *StDofs* with a similar number of introns are highly conserved.

**Figure 3:**
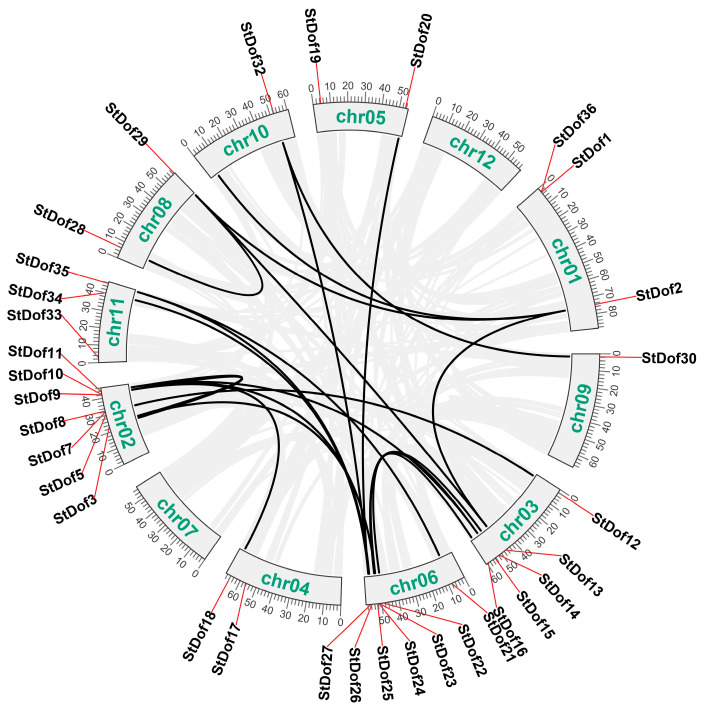
Chromosome distribution and duplication analysis of potato *Dof* genes. Chromosomes 1–12 are shown in a circular form. Black curves denote the details of duplication between potato *Dof* genes.

The 36 *StDof* genes were distributed on ten chromosomes except for chromosomes 7 and 12 (Figure 3; Table 1). The largest number of *StDof* genes were located on chromosome 2, with a total of nine *StDof* genes. Chromosomes 4, 5, 8, and 10 each included two *StDof* genes, while chromosome 9 had just one *StDof* gene. The results of the duplication analysis of *StDofs* revealed that 28 *StDofs* were engaged in 20 segmental duplication events, and these genes were found in all five groups (A, C–F). Aside from segmental duplication, no tandem duplication events were discovered, and eight members of the *StDof* gene were not detected in the duplication events. *StDof1* and *StDof7* were discovered to be involved in two tandem duplication events. Only one member (*StDof1*) was involved in both segmental and tandem duplication (Figure 3). These results suggested that segmental duplication may have played the dominant role in the expansion of the potato *Dof* gene family.

### 2.3. Protein Structure and Conserved Motifs of the StDofs

Ten conserved motifs were identified by the MEME program with unique phylogenetic distributions (Figure 2B). The StDof proteins exhibited a variable number of motifs, ranging from 1 to 7, with a length spanning between 15 and 50 amino acids. Except for *StDof5*, all members of Group A contained seven motifs. Motifs 2, 5, and 7–9 were only present in five members of Group A (Figure 1 and Figure 2B; Supplementary Table S1). By contrast, one motif (Motif 1, Dof domain) can be detected in all 36 StDofs. Similar to other plants, the five CDFs belong to Group A and contain the most conserved motifs.

### 2.4. Profiling of StDof Gene Expression in Different Organs/Tissues

**Figure 4:**
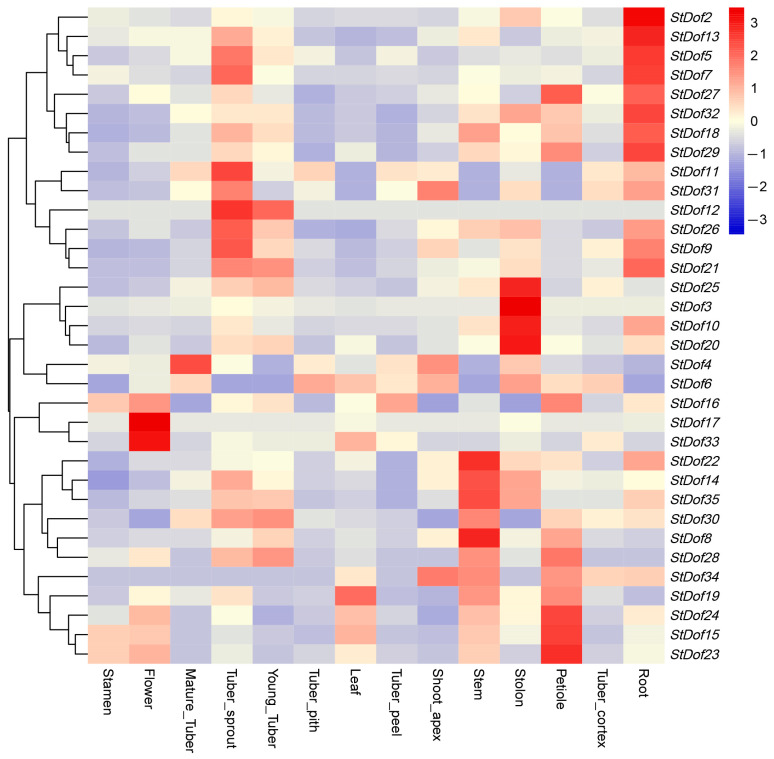
Expression profiles of the *StDof* genes at different developmental stages in specific tissues/organs in potato. Dynamic expression profiles of *StDof* genes for 14 different tissues/organs using publicly available microarray data. Intensity of expression is defined in the colored bar on the right of the chart with red representing increased transcript abundance and blue representing decreased transcript abundance.

By using RNA-seq data (DM 1-3 516 R44-Gene Expression Matrix (TPM)-v6.1), the expression levels of *StDof* genes in 14 different organs/tissues (stamen, flower, mature tuber, young tuber, tuber pith, tuber peel, tuber cortex, shoot apex, stem, petiole, and root) were examined. The results displayed diverse response patterns in different tissues. The *StDofs* can be detected in different tissues and organs, and the expression levels differ in various organs/tissues (Figure 4). For example, the highest expression levels of six genes (*StDof15*, *StDof19*, *StDof20*, *StDof23*, *StDof24*, and *StDof33*) were detected in the leaf, and the other 23 *StDof* genes were highly expressed in the root. The mRNA levels of *StDof17* and *StDof33* were particularly high in flowers, while the expression level of *StDof4* was found to be higher in mature tubers.

### 2.5. Analysis of Cis-Acting Elements in Promoter

**Figure 5:**
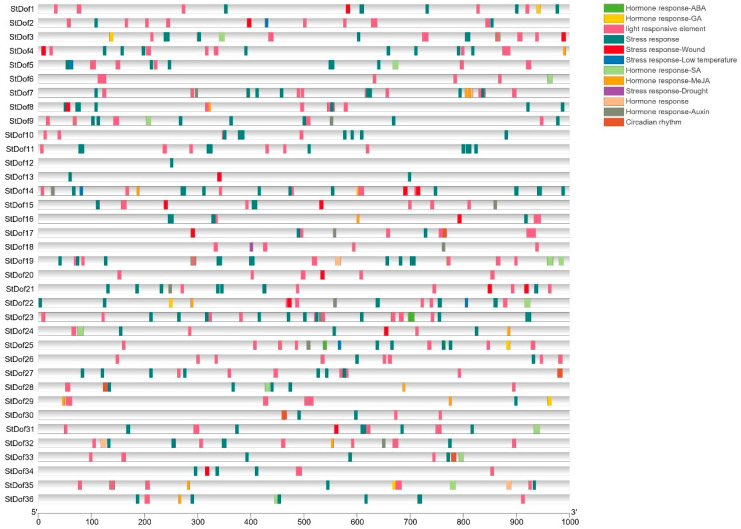
Schematic representation of cis-elements detected in StDof gene promoters using PlantCARE. All identified cis-acting elements were divided into 12 groups (indicated by different colors).

The cis-acting elements within the region of a 1.0 kb sequence upstream from each gene’s ATG site were analyzed using PlantCARE to get a better understanding of the putative regulation mechanism (stress/hormone-related) of *StDofs*. Except for the basic gene expression control elements (CAAT and TATA), 12 groups were divided according to their biological function (Figure 5; Table S2). A variety of hormonal response-related cis-acting elements, such as ABA, MeJA, GA, SA and Auxin, have been discovered. ABA and MeJA are frequently involved in plant stress resistance. Drought- or low-temperature-responsive elements, such as the MYB binding site involved in drought-inducibility (MBS) and low temperature-responsive (LTR) elements. The MYB element was found in *StDof4*/*7*/*9*/*10*/*18*/*19*/*23*. The LTR element was detected in the promoters of *StDof2*/*5*/*14*/*22*/*25*. Other abiotic stress-responsive elements, such as the dehydration-responsive element (DRE), MBS, TC-rich repeats, F-box, ARE and MYC, were also detected in *StDof* gene promoters.

### 2.6. Expression Analysis of StDof Genes under Abiotic/Biotic Stresses and Hormone Treatment

**Figure 6:**
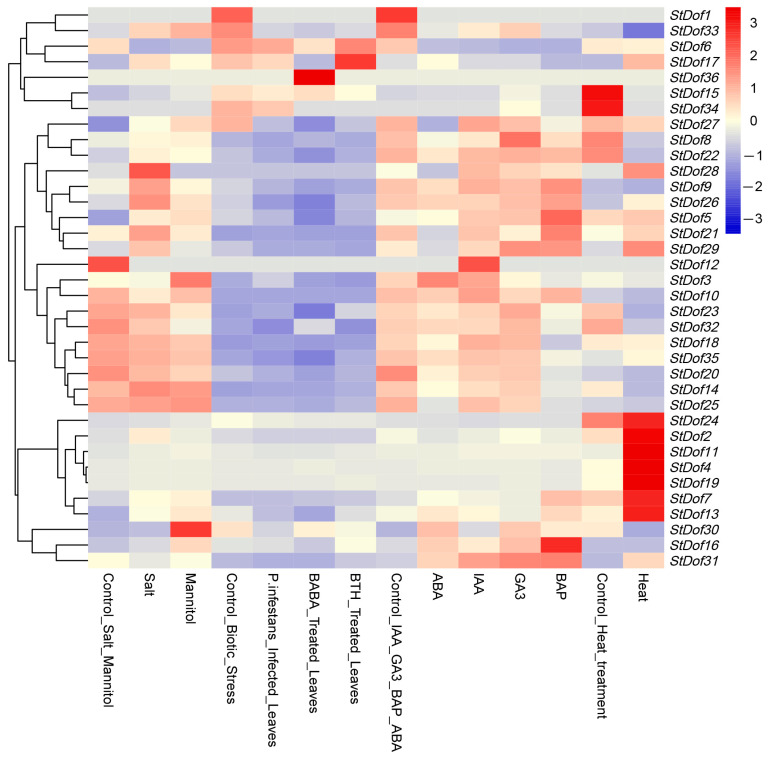
Expression profiles of the *StDof* family genes with multiple treatments in potato. Gene expression was hierarchically clustered. Salt stress, 150mM NaCl; Heat stress, 35 °C; Mannitol, 24 h of 260 µM; BABA and BTH treated 24 h, respectively; *P. infestans* infected leaves 24 h. Up-regulation is indicated by red colors, whereas down-regulation is indicated by blue colors.

The functions of the *StDof* genes in response to abiotic stress remain mostly unclear. To investigate the function of *StDof* genes, their expression patterns under abiotic conditions (salt stress, 150 mM NaCl; heat stress, 35 °C; mannitol, 24 h of 260 µM), biotic stress (BABA and BTH treated 24 h, respectively; *P. infestans* infected leaves 24 h), and hormone treatment (ABA, IAA, GA3, and BAP treated 24 h of 50, 10, 50, and 10 μM, respectively) (DM 1-3 516 R44-Gene Expression Matrix TPM-v6.1) were assessed (Figure 6). The results showed that most *StDofs* genes responded to abiotic stress. Under salt and heat stress, the transcript abundance of eight (*StDof7*/*9*/*13*/*17*/*21*/*26*/*28*/*33*) and eleven (*StDof2*/*4*/*7*/*11*/*13*/*17*/*19*/*24*/*28*/*29*/*31*) *Dof* genes increased. In contrast, salt stress decreased the expression levels of *StDof6* and *StDof12*, while under heat stress, the expression levels of *StDof15* and *StDof34* decreased (Figure 6). After being treated with hormones (24 h of 50 μM ABA, 50 μM GA_3_, 10 μM IAA, and 10 μM BAP), the genes exhibited distinct patterns of expression in response to each specific hormonal treatment. Both *StDof16* and *StDof31* exhibited increased expression levels in all hormone treatments, while the expression levels of *StDof1* and *StDof6* were down-regulated. Some StDof genes also showed variable expression patterns in response to biotic stresses.

### 2.7. Expression Analysis of StDof Genes in Two Potato Cultivars of Contrasting Tolerance under Drought Stress

**Figure 7:**
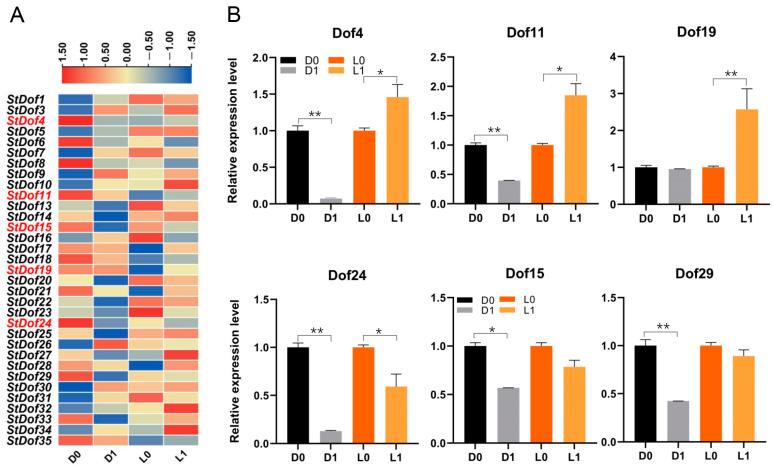
Expression patterns of differentially expressed *Dof* genes in two cultivars. (**A**) RNA-Seq data of differentially expressed *Dof* genes in two cultivars; (**B**) The qRT-PCR validation of differentially expressed *Dof* genes. D0 is the control of DXY and L0 is the control of Long10, D1 and L1 represents DXY and Long10 five days after drought treatment. * *p* < 0.05 and ** *p* < 0.01 (Student’s *t*-test).

Due to differential tolerance to drought stress [39], ‘Long10′ and ‘DXY’ were selected as experimental materials for the expression analysis of *Dof* genes in potato. The expression of all 36 *StDof* genes was tracked using RNA-Seq (six-week-old micropropagated plantlets of ‘Long10′ and ‘DXY’ were exposed to drought stress) to acquire insight into their possible roles in drought stress responses (Figure 7A). According to the findings, drought stress caused significant alterations in the expression profiles of *StDofs*. Drought treatment significantly increased the expression of five *StDof* genes, while it resulted in a decrease in seven other *StDof* genes. Six differentially expressed *StDofs* involved in drought stress response were studied using qRT-PCR to validate the RNA-seq data. The qRT-PCR results agreed with the RNA-seq data (Figure 7; Table S3). Simultaneously, we observed significant differences in the expression levels of *StDofs* (*StCDF1*/*StDof19*, *StCDF2*/*StDof4*, *StCDF3*/*StDof11*, *StCDF4*/*StDof24*, and *StCDF5*/*StDof15*) within two cultivars with differing drought tolerances. These findings suggested that StCDFs were involved in potato drought stress response.

### 2.8. Regulatory Network Mediated by lncRNAs and Their Target Dofs

**Figure 8:**
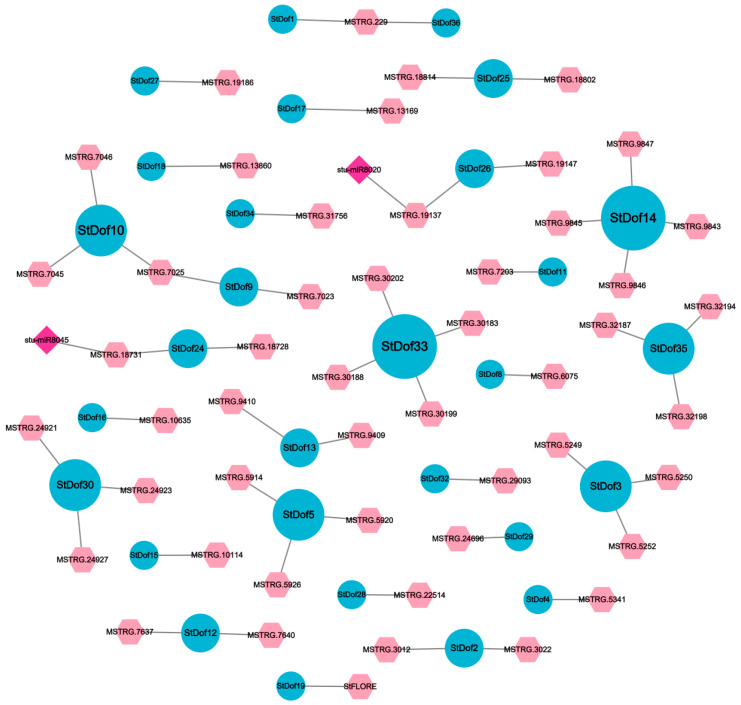
Network diagram of interaction between LncRNA and target *Dof* genes. LncRNA is shown in light red, mRNA is shown in blue, mircroRNA is shown in fuchsia, and the line represents the targeting relationship.

**Figure 9:**
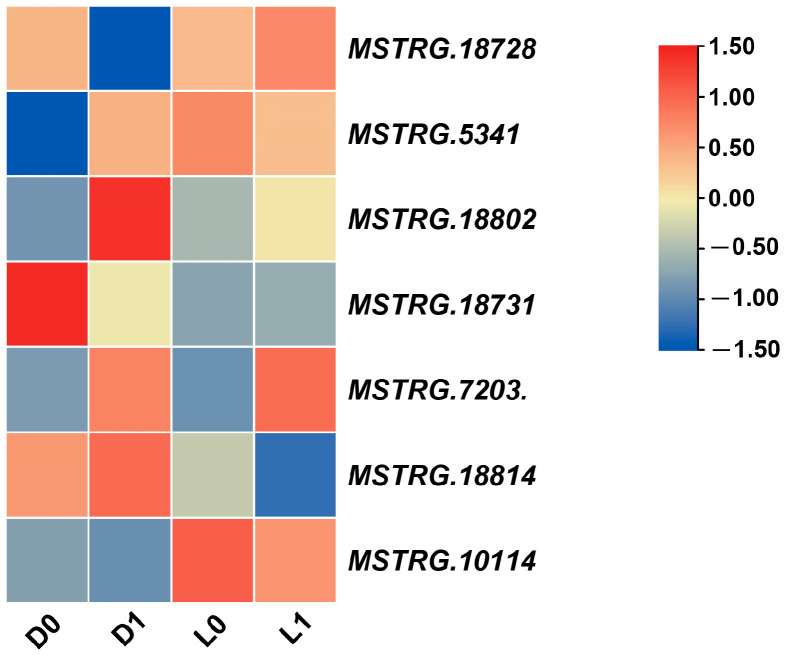
Expression patterns of differentially expressed *lncRNAs* in two cultivars. D0 is the control of DXY and L0 is the control of Long10. D1 and L1 represent DXY and Long10 five days after drought treatment.

Long non-coding RNAs (lncRNAs) operate as cis- or trans-acting regulators of protein-coding genes, playing key roles in plant responses to abiotic stressors. Some Dofs were identified as the targets of lncRNAs in potato—StCDF1/StDof19, together with lncRNA counterpart *StFLORE*; the *StCDF1*–*StFLORE* locus is important for vegetative reproduction and water homeostasis [40]. We proceeded to build regulatory networks mediated by lncRNAs and their target Dofs. In potato, 50 lncRNAs, which target 29 StDofs, were predicted and discovered (Figure 8; Table S4). Notably, *StDof14* and *StDof33* were identified as hub genes in the module, which was cis-regulated by four lncRNAs. Meanwhile, *StDof9*, *StDof25*, and *StDof26* may be regulated by two *lncRNAs*, respectively. Furthermore, two specific micRNAs (*stu-miR8020* and *stu-miR8045*) that were targeted by lncRNAs were also identified. These two micRNAs have previously been reported to associate with the drought stress response [41]. We examined the expression patterns of CDF-related lncRNA in various drought-resistant potato cultivars under drought stress. The results indicated significant differential expression of lncRNA under drought stress (Figure 9), suggesting a potential role in regulating CDF and influencing potato’s response to drought stress.

## 3. Discussion

### 3.1. Identification and Characterization of StDof in Potato

Dof genes are crucial for various plant physiological processes as well as for abiotic and biotic stress responses. A total of 36 *Dof* genes were found in potato. These *StDofs* were categorized into five groups based on the presence of a highly conserved Dof domain in the amino acid sequences (Figure 1 and Figure 3; Table 1) and named in reference to Arabidopsis [42]. The number of *Dof* genes in potato (36) is comparable to that of Arabidopsis (36) and rice (30) [43,44]. Gene duplication occurrences are a major contributor to the gene family’s expansion. In potato, 28 *StDofs* were involved in duplication events (Figure 2), which differs from other potato gene families like the WRKY family [45] and the CIPK [46] family. This implies that duplication is one of the most important processes contributing to the expansion of the potato *Dof* gene family.

Interestingly, the analysis of conserved motifs revealed a series of motifs that were largely confined to a single phylogenetic Group A of Dofs (as the CDF group, Figure 1 and Figure 3). CDF proteins often function as important regulators, along with other proteins, through a special motif from the C-terminal of Dof proteins [6,13,14,47]. Most StDofs contain Motif 1 (Dof domain); however, only the CDF group contains many conserved motifs, with some encompassing up to seven motifs. Indeed, most CDFs are known to work downstream of GI, regulating responses to stress and flowering [6,31]. For example, Motif 2 is a GI- and FKF1-binding domain (Figure 3) [6,47], Motif 9 is a predicted nuclear localization signal, and Motif 5 is an N-myristoylation site [48]; these motifs only exist in the CDFs (Figure 1 and Figure 3). These results suggest that CDF-related Dofs play more extensive regulatory roles in plant growth, development, and stress response than other groups with fewer motifs. This may indicate that these StDof proteins play an equally important role in potato.

### 3.2. Specific StDofs Involved in Response to Drought Stress in Potato

Previous studies have shown that *Dof* genes are involved a wide range of stress responses [33,47]. Our findings indicated that the expression of *StDofs* was extensively responsive to a variety of bio- and abiotic stresses, as well as to hormones, suggesting that StDofs may be involved in multiple abiotic stress responses in potato. As one of the major abiotic stresses affecting plant growth and development, drought stress seriously affects potato yield and quality. When comparing drought-tolerant (Long10) and drought-sensitive (DXY) cultivars, the results showed that Dof TFs were differentially expressed in potato varieties (Figure 7). Due to the important regulatory role of CDF, we also identified potato CDF, StCDF1/StDof19, StCDF2/StDof4, StCDF3/StDof11, StCDF4/StDof24, and StCDF5/StDof15, which are homologs of Arabidopsis CDFs [6,14].

Based on their nature (tolerant or sensitive to drought) and drought treatment, the genotypes were grouped into similar groups, displaying different expression patterns for control and stress (Figure 7). Like *StCDF1*, its ortholog in tomato *SlCDF1* demonstrated increased drought and salt tolerance [47]. In Arabidopsis, overexpression of the orthologs gene *AtCDF3* improved the tolerance of the transgenic plant to several abiotic stresses (drought, cold, and osmotic stress) by modulating the CBF/DREB system [32,33]. In potato, StCDF1 was identified as a major-effect quantitative trait locus for plant maturity and the initiation of tuber development. It was also shown that StCDF1, together with the lncRNA *StFLORE*, regulates water loss by affecting stomatal growth and diurnal opening [14,40]. In our results, *StCDF1* was significantly up-regulated in the drought-resistant cultivar ‘Long10’, but not in the drought-sensitive cultivar ‘DXY’. Meanwhile, the expression levels of *StCDF2* and *StCDF3* were reduced in the drought-sensitive cultivar ‘DXY’, but were dramatically increased in the drought-resistant cultivar ‘Long10’. As a result, research shows that potato StCDFs may play a crucial role in drought and other stress responses.

### 3.3. The lncRNA-Mediated Regulatory Pathways Involved in Response to Drought Stress

LncRNAs are primarily involved in the epigenetic and transcriptional regulation of gene transcription and expression [49]. LncRNAs have been confirmed to be related to stress in various plants, and several studies have explored the relationship between lncRNAs and mRNAs at a genome-wide level [50,51]. In rice, a total of 191 lncRNAs, 2115 mRNAs, and 32 miRNAs (microRNAs) were found to be differentially expressed under drought stress [52]. The lncRNA *Auxin-regulated Promoter Loop* (*AtAPOLO*) interacted with the TF WRKY42 to trigger the cell expansion of root hair in response to cold [53]. In response to cotton salt stress, *LncRNA973* regulated reactive oxygen species-scavenging genes, transcription factors, and genes associated with salt stress [54]. In this paper, we predicted that lncRNAs regulated 26 *Dofs*, and 10 of them were regulated by multiple lncRNAs (Figure 8). Recently, lncRNA *StFLORE* has been reported to regulate water loss by affecting stomatal growth and diurnal opening by negatively regulating its target gene *StCDF1*/*StDof19* in potato [40]. LncRNAs function as regulators of gene transcription and expression, and they also engage in interactions with miRNAs [55,56]. In this paper, we also identified the target miRNAs of two lncRNAs. like Stu-miR8045-lncRNA-StCDF4. Collectively, we highlight a unique role of Dofs in stress that integrates potato’s response to adverse environmental conditions with different aspects of potato growth and development.

## 4. Materials and Methods

### 4.1. Identification of Dof Genes in Potato

Dof proteins were identified by Hidden Markov Models (HMM, e value < 0.01) (PF02701) in the Pfam database (http://pfam.sanger.ac.uk/, accessed on 18 October 2022) and a search against the proteins of the potato reference genome by BLAST (e value < 1 × 10^−5^), the potato genome database (http://solanaceae.plantbiology.msu.edu/pgsc) based on Dof genes from Arabidopsis and rice [42], Malus domestica [43], sorghum [44], and grape [8]. The candidate members were confirmed by a Conserved Domain Data (CDD) search (http://www.ncbi.nlm.nih.gov/structure/cdd/wrpsb.cgi, accessed on 19 October 2022).

### 4.2. Phylogenetic and Structural Analysis of StDof Genes

MEGA7.0 (Pennsylvania, USA) and Clustal X (Dublin, Ireland) were used to perform a phylogenetic analysis and amino acid sequences alignment. The phylogenetic tree was built using the neighbor-joining statistical approach and 1000 bootstrap replications. Domain analysis was performed by SMART (http://smart.embl-heidelberg.de/, accessed on 22 October 2022). Gene Structure Display Server 2.0 (GSDS) (http://gsds.cbi.pku.edu.cn, accessed on 22 October 2022) was used to create the gene structure. The annotated potato genome was used to obtain information on the introns and exons of the *StDofs*.

### 4.3. Conserved Motifs and Gene Location and Duplication Analysis of StDof Genes

Conserved motifs were identified by MEME (https://meme-suite.org/meme/tools/meme, accessed on 25 October 2022). The *Dof* genes’ locations were obtained from the potato gene model annotation files v6.1 (http://spuddb.uga.edu, accessed on 25 October 2022). The entire protein sequences were subjected to duplication studies using BLAST (all vs. all), and the duplications were detected using Mcscan X (e-value 1 × 10^−5^ and on the nearby gene loci).

### 4.4. Cis-Acting Element of Promoter Analysis

The potato genome database (http://solanaceae.plantbiology.msu.edu/pgsc, accessed on 18 October 2022) was used to obtain the promoter regions of *StDofs*. Using the PlantCARE databases (http://bioinformatics.psb.ugent.be/webtools/plantcare, accessed on 3 January 2023), we searched the 1.0 kb region upstream of the transcription start site (ATG) to identify cis-acting regulatory elements.

### 4.5. Microarray Data Analysis

Microarray data were used for further analysis in order to comprehend the geographical and temporal expression patterns of *StDof* genes. Using publicly available information (DM 1–3 516 R44-Gene Expression Matrix (TPM)-v6.1, http://spuddb.uga.edu/dm_v6_1_download.shtml, accessed on 18 October 2023), the expression profiles of *StDofs* in various tissues and with different treatments were investigated. A heatmap was constructed using the Pheatmap (https://cran.rproject.org/web/packages/pheatmap/, accessed on 26 October 2023) package of R.

### 4.6. Plant Materials and Treatments

Potato (*Solanum tuberosum* L.) cultivars ‘Long10′ (drought tolerant) and ‘DXY’ (drought susceptible) were used in the experiment [39]. The potato tubers, which weighed around 150 g, were collected and grown in vermiculite pots of 4 L in a greenhouse with natural light at a temperature of 25 ± 2 °C. The seedlings were given weekly applications of a complete fertilizer solution and water after sprouting. Drought treatments were carried out on the treatment samples once the plants had grown for 25 days. The soil water content in the pots was monitored two times every day using TDR-300 sensors (Spectrum R, Aurora, IL, USA). The soil water content in the pots was kept at 75–80% in the control group (D0 and L0). When the water content of the soil in the pots had decreased to 35–40%, it was considered the first day of drought treatment. Samples were collected after a 5-day drought treatment, and three replicates (each containing 10 plants) were employed [46,57].

### 4.7. Quantitative RT-PCR (qRT-PCR) Analysis

IQ SYBR Green Supermix (Bio-Rad, California, America) was used for qRT-PCR. For each sample, three biological replicates, each comprising three technical replicates, were tested. The gene expression levels were normalized against the *StEF1a* (Soltu.DM.06G005680) gene. The relative expression levels were calculated using the 2^−ΔΔCt^ method.

### 4.8. Statistical Analysis

SPSS 20.0 (SPSS, Armonk, NY, USA) was used for significant difference analysis. The experiment was performed in three biological replicates; data are mean values ± SE of three biological replicates.

## 5. Conclusions

In conclusion, the functional characterization of StDofs will benefit future research into the mechanism of abiotic stress tolerance (drought, salt, and cold, etc.). The findings also suggest several StCDFs as potential candidates for enhancing abiotic stress resistance in potatoes. This provides new insights into the Dof gene family’s evolutionary and functional divergence, which can benefit gene functional investigations of prospective Dof genes, providing an essential genetic resource for future studies including multiple gene knockouts and protein–protein interactions. LncRNAs are a class of heterogeneous regulatory transcripts that play a variety of regulatory roles in plant development and stress response. To date, only one report has reported a link between CDFs and lncRNA. Therefore, it is important to determine whether functional roles between Dofs and lncRNA are more widespread than currently known. Therefore, additional studies are required to fully understand the molecular mechanisms by which Dofs orchestrate metabolic homeostasis, stress responses, crop improvement, and plant growth and development. First, the key regulatory genes were mined through the identification and screening of core germplasm resources, and the molecular biological basis of character differences was analyzed. The use of genomics and molecular biology and other modern technological means has the potential to improve the genetic efficiency of crops, improving or innovating crop varieties, through the organic combination of biological breeding and conventional breeding, including genetic engineering.

## Figures and Tables

**Table 1:** Features of Dof gene family in *S. tuberosum*.

Gene ID	Gene Symbol	Group	Chromosome	Start	End	Gene Length (bp)	Protein Length (aa)	NCBI Accession
Soltu.DM.01G002740	*StDof1*	C	01	2,885,950	2,886,891	819	272	-
Soltu.DM.01G035240	*StDof2*	C	01	74,752,808	74,755,079	852	283	XP_006339731.1
Soltu.DM.02G008570	*StDof3*	D	02	23,280,583	23,279,690	585	194	XP_006367424.1
Soltu.DM.02G009620	*StDof4*	A	02	24,499,789	24,496,055	1398	465	XP_006363404.1
Soltu.DM.02G016040	*StDof5*	A	02	30,575,387	30,576,594	498	165	XP_006362415.1
Soltu.DM.02G017380	*StDof6*	E	02	31,859,700	31,861,685	1197	398	XP_006347015.1
Soltu.DM.02G017390	*StDof7*	F	02	31,876,798	31,874,957	858	285	XP_006347016.1
Soltu.DM.02G018030	*StDof8*	E	02	32,428,493	32,429,942	849	282	XP_006347078.1
Soltu.DM.02G029480	*StDof9*	E	02	42,036,367	42,037,640	843	280	XP_006338231.1
Soltu.DM.02G029580	*StDof10*	D	02	42,088,877	42,087,455	723	240	XP_006338240.1
Soltu.DM.02G031630	*StDof11*	A	02	43,678,538	43,675,014	1410	469	XP_006338444.1
Soltu.DM.03G001980	*StDof12*	E	03	1,907,551	1,908,434	777	258	XP_015162392.
Soltu.DM.03G022110	*StDof13*	C	03	46,929,019	46,927,517	786	261	XP_006350511.1
Soltu.DM.03G026810	*StDof14*	F	03	51,573,013	51,570,712	993	330	XP_006344507.1
Soltu.DM.03G030070	*StDof15*	A	03	54,420,292	54,417,525	1512	503	XP_006364590.1
Soltu.DM.03G036130	*StDof16*	E	03	59,179,733	59,179,107	627	208	XP_006343007.1
Soltu.DM.04G026220	*StDof17*	C	04	56,550,622	56,549,822	801	266	XP_015158674.1
Soltu.DM.04G034500	*StDof18*	D	04	65,958,711	65,957,509	717	238	XP_015167546.1
Soltu.DM.05G005140	*StDof19*	A	05	4,485,531	4,488,495	1329	442	XP_006355111.1
Soltu.DM.05G025040	*StDof20*	E	05	53,480,945	53,479,248	909	302	XP_006346013.1
Soltu.DM.06G005290	*StDof21*	F	06	8,124,967	8,122,936	942	313	XP_006366600.2
Soltu.DM.06G022770	*StDof22*	E	06	49,163,821	49,167,835	1131	376	KAH0749417.1
Soltu.DM.06G025370	*StDof23*	E	06	51,314,599	51,316,145	960	319	XP_015167508.1
Soltu.DM.06G025850	*StDof24*	A	06	51,760,526	51,757,714	1479	492	XP_006354775.1
Soltu.DM.06G026620	*StDof25*	F	06	52,433,441	52,435,513	843	280	XP_006347454.1
Soltu.DM.06G030750	*StDof26*	F	06	55,730,228	55,727,997	909	302	XP_006365745.1
Soltu.DM.06G031420	*StDof27*	E	06	56,312,496	56,309,962	963	320	-
Soltu.DM.08G004890	*StDof28*	C	08	6,182,848	6,184,359	756	251	XP_006352301.2
Soltu.DM.08G029850	*StDof29*	C	08	58,857,292	58,860,058	999	332	XP_006344006.1
Soltu.DM.09G002280	*StDof30*	E	09	1,781,087	1,778,703	1212	403	XP_006341037.1
Soltu.DM.10G005360	*StDof31*	C	10	4,885,761	4,883,678	816	271	XP_006362132.1
Soltu.DM.10G020610	*StDof32*	E	10	52,237,955	52,239,789	1125	374	XP_006349511.1
Soltu.DM.11G002690	*StDof33*	E	11	2,808,209	2,810,610	1140	379	XP_006353273.1
Soltu.DM.11G020390	*StDof34*	E	11	40,267,218	40,265,223	936	311	KAH0712769.1
Soltu.DM.11G025780	*StDof35*	F	11	45,731,877	45,733,968	909	302	XP_006366395.1
Soltu.DM.01G002730	*StDof36*	C	01	2,882,119	2,881,166	954	317	KAH0724332.1

## Data Availability

Data are contained within the article and Supplementary Materials.

## Supplementary Materials

The following supporting information can be downloaded at: https://www.mdpi.com/article/10.3390/ijms25063488/s1.

## References

[1] Lindemose S., O’Shea C., Jensen M.K., Skriver K. (2013). Structure, function and networks of transcription factors involved in abiotic stress responses. Int. J. Mol. Sci..

[2] Manna M., Thakur T., Chirom O., Mandlik R., Deshmukh R., Salvi P. (2021). Transcription factors as key molecular target to strengthen the drought stress tolerance in plants. Physiol. Plant..

[3] Yanagisawa S. (2004). Dof Domain Proteins: Plant-Specific Transcription Factors Associated with Diverse Phenomena Unique to Plants. Plant Cell Physiol..

[4] Wang P., Yan Z., Zong X., Yan Q., Zhang J. (2021). Genome-wide analysis and expression profiles of the Dof family in Cleistogenes songorica under temperature, salt and ABA treatment. Plants.

[5] Khan I., Khan S., Zhang Y., Zhou J. (2021). Genome-wide analysis and functional characterization of the Dof transcription factor family in rice (*Oryza sativa* L.). Planta.

[6] Renau-Morata B., Carrillo L., Dominguez-Figueroa J., Vicente-Carbajosa J., Molina R.V., Nebauer S.G., Medina J. (2020). CDF transcription factors: Plant regulators to deal with extreme environmental conditions. J. Exp. Bot..

[7] Huang Y., Han Z., Cheng N., Luo M., Bai X., Xing Y. (2019). Minor Effects of 11 Dof Family Genes Contribute to the Missing Heritability of Heading Date in Rice (*Oryza sativa* L.). Front. Plant Sci..

[8] Wang Z., Wang Y., Tong Q., Xu G., Xu M., Li H., Fan P., Li S. (2021). Transcriptomic analysis of grapevine Dof transcription factor gene family in response to cold stress and functional analyses of the VaDof17d gene. Planta.

[9] Krohn N.M., Yanagisawa S., Grasser K.D. (2002). Specificity of the stimulatory interaction between chromosomal HMGB proteins and the transcription factor Dof2 and its negative regulation by protein kinase CK2-mediated phosphorylation. J. Biol. Chem..

[10] Zhang B., Chen W., Foley R.C., Büttner M., Singh K.B. (1995). Interactions between distinct types of DNA binding proteins enhance binding to ocs element promoter sequences. Plant Cell.

[11] Vicente-Carbajosa J., Moose S.P., Parsons R.L., Schmidt R.J. (1997). A maize zinc-finger protein binds the prolamin box in zein gene promoters and interacts with the basic leucine zipper transcriptional activator Opaque2. Proc. Natl. Acad. Sci. USA.

[12] Diaz I., Vicente-Carbajosa J., Abraham Z., Martinez M., Isabel-La Moneda I., Carbonero P. (2002). The GAMYB protein from barley interacts with the DOF transcription factor BPBF and activates endosperm-specific genes during seed development. Plant J..

[13] Imaizumi T., Schultz T.F., Harmon F.G., Ho L.A., Kay S.A. (2005). FKF1 F-box protein mediates cyclic degradation of a repressor of CONSTANS in *Arabidopsis*. Science.

[14] Kloosterman B., Abelenda J.A., Gomez Mdel M., Oortwijn M., de Boer J.M., Kowitwanich K., Horvath B.M., van Eck H.J., Smaczniak C., Prat S. (2013). Naturally occurring allele diversity allows potato cultivation in northern latitudes. Nature.

[15] Elrouby N., Bonequi M.V., Porri A., Coupland G. (2013). Identification of *Arabidopsis* SUMO-interacting proteins that regulate chromatin activity and developmental transitions. Proc. Natl. Acad. Sci. USA.

[16] da Silva D.C., da Silveira Falavigna V., Fasoli M., Buffon V., Porto D.D., Pappas G.J., Pezzotti M., Pasquali G., Revers L.F. (2016). Transcriptome analyses of the Dof-like gene family in grapevine reveal its involvement in berry, flower and seed development. Hortic. Res..

[17] Liu X., Liu Z., Hao Z., Chen G., Qi K., Zhang H., Jiao H., Wu X., Zhang S., Wu J. (2020). Characterization of Dof family in *Pyrus bretschneideri* and role of PbDof9.2 in flowering time regulation. Genomics.

[18] Zhang L., Jiang A., Thomson G., Kerr-Phillips M., Phan C., Krueger T., Jaudal M., Wen J., Mysore K.S., Putterill J. (2019). Overexpression of Medicago MtCDFd1_1 Causes Delayed Flowering in Medicago via Repression of MtFTa1 but Not MtCO-Like Genes. Front. Plant Sci..

[19] Rymen B., Kawamura A., Schafer S., Breuer C., Iwase A., Shibata M., Ikeda M., Mitsuda N., Koncz C., Ohme-Takagi M. (2017). ABA Suppresses Root Hair Growth via the OBP4 Transcriptional Regulator. Plant Physiol..

[20] Yang J., Yang M.F., Wang D., Chen F., Shen S.H. (2010). JcDof1, a Dof transcription factor gene, is associated with the light-mediated circadian clock in *Jatropha curcas*. Physiol. Plant..

[21] Zhuo M., Sakuraba Y., Yanagisawa S. (2020). A Jasmonate-Activated MYC2-Dof2.1-MYC2 Transcriptional Loop Promotes Leaf Senescence in *Arabidopsis*. Plant Cell.

[22] Chen P., Yan M., Li L., He J., Zhou S., Li Z., Niu C., Bao C., Zhi F., Ma F. (2020). The apple DNA-binding one zinc-finger protein MdDof54 promotes drought resistance. Hortic. Res..

[23] Li G., Xu W., Jing P., Hou X., Fan X. (2021). Overexpression of VyDOF8, a Chinese wild grapevine transcription factor gene, enhances drought tolerance in transgenic tobacco. Environ. Exp. Bot..

[24] Qin H., Wang J., Chen X., Wang F., Peng P., Zhou Y., Miao Y., Zhang Y., Gao Y., Qi Y. (2019). Rice OsDOF15 contributes to ethylene-inhibited primary root elongation under salt stress. New Phytol..

[25] Guo T., Wang S., Zhang T., Xu L., Li Y., Chao Y., Han L. (2021). Expression of the *Medicago truncatula* MtDof32 transcription factor regulates plant growth and enhances abiotic stress tolerances in transgenic *Arabidopsis*. Environ. Exp. Bot..

[26] Yang Q., Yang Q., Chen Q., Zhu Y. (2018). Identification of MdDof genes in apple and analysis of their response to biotic or abiotic stress. Funct Plant Biol..

[27] Xu J., Dai H. (2016). Brassica napus Cycling Dof Factor1 (BnCDF1) is involved in flowering time and freezing tolerance. Plant Growth Regul..

[28] Yu Y.H., Bian L., Wan Y.T., Jiao Z.L., Guo D.L. (2019). Grape (*Vitis vinifera*) VvDOF3 functions as a transcription activator and enhances powdery mildew resistance. Plant Physiol. Biochem..

[29] Nan H., Ludlow R.A., Lu M., An H. (2021). Genome-Wide Analysis of Dof Genes and Their Response to Abiotic Stress in Rose (*Rosa chinensis*). Front. Genet..

[30] Yang G., Gao X., Ma K., Li D., Jia C., Zhai M., Xu Z. (2018). The walnut transcription factor JrGRAS2 contributes to high temperature stress tolerance involving in Dof transcriptional regulation and HSP protein expression. BMC Plant Biol..

[31] Fornara F., de Montaigu A., Sanchez-Villarreal A., Takahashi Y., Ver Loren van Themaat E., Huettel B., Davis S.J., Coupland G. (2015). The GI-CDF module of *Arabidopsis* affects freezing tolerance and growth as well as flowering. Plant J..

[32] Corrales A.R., Carrillo L., Lasierra P., Nebauer S.G., Dominguez-Figueroa J., Renau-Morata B., Pollmann S., Granell A., Molina R.V., Vicente-Carbajosa J. (2017). Multifaceted role of cycling DOF factor 3 (CDF3) in the regulation of flowering time and abiotic stress responses in *Arabidopsis*. Plant Cell Environ..

[33] Renau-Morata B., Molina R.V., Carrillo L., Cebolla-Cornejo J., Sanchez-Perales M., Pollmann S., Dominguez-Figueroa J., Corrales A.R., Flexas J., Vicente-Carbajosa J. (2017). Ectopic Expression of CDF3 Genes in Tomato Enhances Biomass Production and Yield under Salinity Stress Conditions. Front. Plant Sci..

[34] Tominaga Y., Suzuki K., Uemura M., Kawamura Y. (2021). In Planta Monitoring of Cold-Responsive Promoter Activity Reveals a Distinctive Photoperiodic Response in Cold Acclimation. Plant Cell Physiol..

[35] Molina-Hidalgo F.J., Medina-Puche L., Canete-Gomez C., Franco-Zorrilla J.M., Lopez-Vidriero I., Solano R., Caballero J.L., Rodriguez-Franco A., Blanco-Portales R., Munoz-Blanco J. (2017). The fruit-specific transcription factor FaDOF2 regulates the production of eugenol in ripe fruit receptacles. J. Exp. Bot..

[36] Song A., Gao T., Li P., Chen S., Guan Z., Wu D., Xin J., Fan Q., Zhao K., Chen F. (2016). Transcriptome-Wide Identification and Expression Profiling of the DOF Transcription Factor Gene Family in Chrysanthemum morifolium. Front. Plant Sci..

[37] Ewas M., Khames E., Ziaf K., Shahzad R., Nishawy E., Ali F., Subthain H., Amar M.H., Ayaad M., Ghaly O. (2017). The Tomato DOF Daily Fluctuations 1, TDDF1 acts as flowering accelerator and protector against various stresses. Sci. Rep..

[38] Su Y., Liang W., Liu Z., Wang Y., Zhao Y., Ijaz B., Hua J. (2017). Overexpression of GhDof1 improved salt and cold tolerance and seed oil content in *Gossypium hirsutum*. J. Plant Physiol..

[39] Yu B., Yang H., Wang L., Liu Y., Bai J., Zhang F., Wang D., Zhang J. (2018). Relationship between potato canopy-air temperature difference and drought tolerance. Acta Agron. Sin..

[40] Ramirez G.L., Shi L., Bergonzi S.B., Oortwijn M., Franco-Zorrilla J.M., Solano-Tavira R., Visser R.G.F., Abelenda J.A., Bachem C.W.B. (2021). Potato CYCLING DOF FACTOR 1 and its lncRNA counterpart StFLORE link tuber development and drought response. Plant J..

[41] Zhang N., Yang J., Wang Z., Wen Y., Wang J., He W., Liu B., Si H., Wang D. (2014). Identification of novel and conserved microRNAs related to drought stress in potato by deep sequencing. PLoS ONE.

[42] Lijavetzky D., Carbonero P., Vicente-Carbajosa J. (2003). Genome-wide comparative phylogenetic analysis of the rice and *Arabidopsis* Dof gene families. BMC Evol. Biol..

[43] Zhang Z., Yuan L., Liu X., Chen X., Wang X. (2018). Evolution analysis of Dof transcription factor family and their expression in response to multiple abiotic stresses in *Malus domestica*. Gene.

[44] Kushwaha H., Gupta S., Singh V.K., Rastogi S., Yadav D. (2011). Genome wide identification of Dof transcription factor gene family in sorghum and its comparative phylogenetic analysis with rice and *Arabidopsis*. Mol. Biol. Rep..

[45] Zhang C., Wang D., Yang C., Kong N., Shi Z., Zhao P., Nan Y., Nie T., Wang R., Ma H. (2017). Genome-wide identification of the potato WRKY transcription factor family. PLoS ONE.

[46] Ma R., Liu W., Li S., Zhu X., Yang J., Zhang N., Si H. (2021). Genome-Wide Identification, Characterization and Expression Analysis of the CIPK Gene Family in Potato (*Solanum tuberosum* L.) and the Role of StCIPK10 in Response to Drought and Osmotic Stress. Int. J. Mol. Sci..

[47] Corrales A.R., Nebauer S.G., Carrillo L., Fernandez-Nohales P., Marques J., Renau-Morata B., Granell A., Pollmann S., Vicente-Carbajosa J., Molina R.V. (2014). Characterization of tomato Cycling Dof Factors reveals conserved and new functions in the control of flowering time and abiotic stress responses. J. Exp. Bot..

[48] Noguero M., Atif R.M., Ochatt S., Thompson R.D. (2013). The role of the DNA-binding One Zinc Finger (DOF) transcription factor family in plants. Plant Sci..

[49] Li S., Cheng Z., Dong S., Li Z., Zou L., Zhao P., Guo X., Bao Y., Wang W., Peng M. (2022). Global identification of full-length cassava lncRNAs unveils the role of cold-responsive intergenic lncRNA 1 in cold stress response. Plant Cell Environ..

[50] Gong H., You J., Zhang X., Liu Y., Zhao F., Cui X., Zhang Y. (2021). Genome-Wide Identification and Functional Analysis of Long Non-coding RNAs in Sesame Response to Salt Stress. J. Plant Biol..

[51] Kumar N., Bharadwaj C., Sahu S., Shiv A., Shrivastava A.K., Reddy S.P.P., Soren K.R., Patil B.S., Pal M., Soni A. (2021). Genome-wide identification and functional prediction of salt- stress related long non-coding RNAs (lncRNAs) in chickpea (*Cicer arietinum* L.). Physiol. Mol. Biol. Plants.

[52] Yang X., Liu C., Niu X., Wang L., Li L., Yuan Q., Pei X. (2022). Research on lncRNA related to drought resistance of Shanlan upland rice. BMC Genom..

[53] Moison M., Martínez Pacheco J., Lucero L., Fonouni-Farde C., Rodríguez-Melo J., Mansilla N., Christ A., Bazin J., Benhamed M., Ibañez F. (2021). The lncRNA APOLO interacts with the transcription factor WRKY42 to trigger root hair cell expansion in response to cold. Mol. Plant.

[54] Zhang X., Dong J., Deng F., Wang W., Cheng Y., Song L., Hu M., Shen J., Xu Q., Shen F. (2019). The long non-coding RNA lncRNA973 is involved in cotton response to salt stress. BMC Plant Biol..

[55] Zhang X., Shen J., Xu Q., Dong J., Song L., Wang W., Shen F. (2021). Long noncoding RNA lncRNA354 functions as a competing endogenous RNA of miR160b to regulate ARF genes in response to salt stress in upland cotton. Plant Cell Environ..

[56] He X., Guo S., Wang Y., Wang L., Shu S., Sun J. (2020). Systematic identification and analysis of heat-stress-responsive lncRNAs, circRNAs and miRNAs with associated co-expression and ceRNA networks in cucumber (*Cucumis sativus* L.). Physiol. Plant..

[57] Yang L., Zhang N., Wang K., Zheng Z., Wei J., Si H. (2023). CBL-Interacting Protein Kinases 18 (CIPK18) Gene Positively Regulates Drought Resistance in Potato. Int. J. Mol. Sci..

