# Supplementary material for: DNA-Binding with One Finger (Dof) Transcription Factor Gene Family Study Reveals Differential Stress-Responsive Transcription Factors in Contrasting Drought Tolerance Potato Species
Source: Int J Mol Sci. 2024 Mar 20;25(6):3488. doi: 10.3390/ijms25063488 (PMC10970974; doi:10.3390/ijms25063488)
Supplement: Supplementary file 1 [file ijms-25-03488-s001.zip › Table S1.pdf]

Table S1. The conserved motifs of 36 StDofs in potato

Motif Name	Motif logo	E-Value	Site Count	Width	Possible Motif
Motif 1	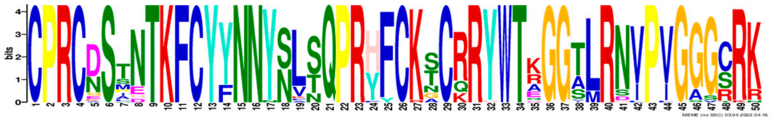	9.3e-1633	36	50	CPRCDSANTKFCYYNNYSLTQPRYFCKSCRR[illegible]<br>YWTKGGTLRNVPVGGGCRK
Motif 2	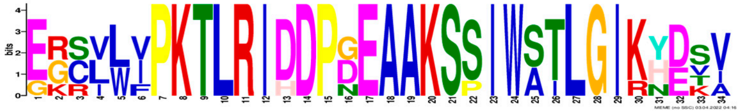	6.7e-067	5	34	EKCLWVPKTLRIDDPGEAAKSSIWATLGIKHDTV
Motif 3	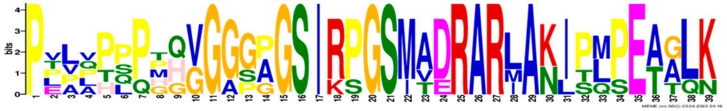	1.8e-041	5	39	PVVVPLQPHGGGGAGSIRPGSMADRARMANIPMPETALK
Motif 4	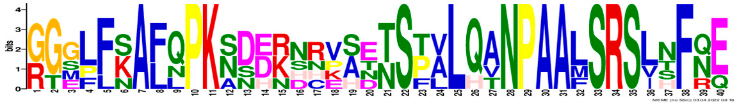	4.5e-034	5	40	GGSPFSAFQPKNDDNNRVSENSTVLQANPAALSRSVNFNE
Motif 5	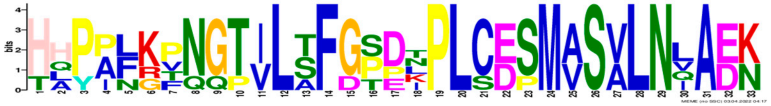	4.5e-033	5	33	HHPAFKPNGTVLSFGPDLPLCDSMASVLNLAEN
Motif 6	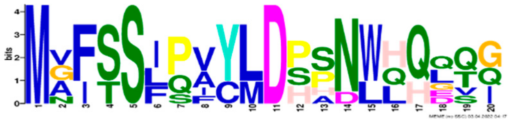	3.4e-027	7	20	MAFSSIPFYLDPPNWHHEQG

Motif 7	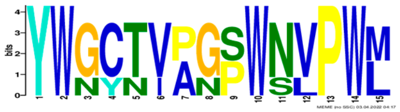	3.7e-016	4	15	YWGCTIPGSWNVPWM
Motif 8	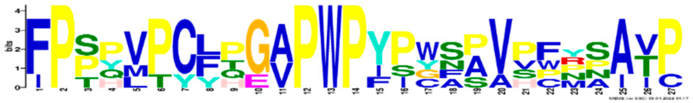	5.6e-020	5	27	FPPYMTCYPGAPWPYPCSPVPWNSAVP
Motif 9	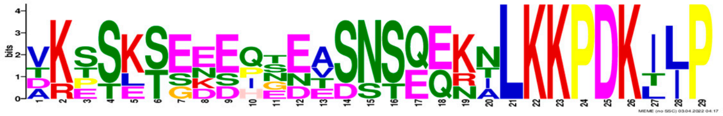	1.8e-016	5	29	VKSSKTEDDQNDASNTQQKTLKKPDKILP
Motif 10	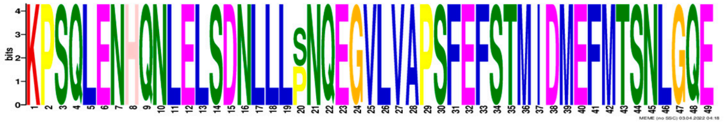	8.1e-014	2	49	KPSQLENHQNLELSDNLLLPNQEGVLVAPS FEFSTMIDMEFMTSNLGQE
