# Supplementary material for: DNA-Binding with One Finger (Dof) Transcription Factor Gene Family Study Reveals Differential Stress-Responsive Transcription Factors in Contrasting Drought Tolerance Potato Species
Source: Int J Mol Sci. 2024 Mar 20;25(6):3488. doi: 10.3390/ijms25063488 (PMC10970974; doi:10.3390/ijms25063488)
Supplement: Supplementary file 1 [file ijms-25-03488-s001.zip › Table S2-S4.pdf]

Table S2. The information of *cis*-element in promoter region including sequence and function.

**Respose of type**	***Cis*-element**	**Sequence**	**Function of *cis* -element**
Circadian rhythm	circadian	CAAAGATATC	cis-acting regulatory element involved in circadian control
Hormone response-ABA	ABRE	AACCCGG	cis-acting element involved in the abscisic acid responsiveness
		ACGTG	cis-acting element involved in the abscisic acid responsiveness
		CACGTG	cis-acting element involved in the abscisic acid responsiveness
		CGCACGTGTC	cis-acting element involved in the abscisic acid responsiveness
		CGTACGTGCA	cis-acting element involved in the abscisic acid responsiveness
		GACACGTGGC	cis-acting element involved in the abscisic acid responsiveness
		TACGGTC	cis-acting element involved in the abscisic acid responsiveness
	ABRE3a	TACGTG	cis-acting element involved in the abscisic acid responsiveness
	ABRE4	CACGTA	cis-acting element involved in the abscisic acid responsiveness
Hormone response-Auxin	AuxRR-core	GGTCCAT	cis-acting regulatory element involved in auxin responsiveness
	TGA-element	AACGAC	auxin-responsive element
Hormone response-GA	GARE-motif	TCTGTTG	gibberellin-responsive element
	P-box	CCTTTTG	gibberellin-responsive element
Hormone response-MeJA	CGTCA-motif	CGTCA	cis-acting regulatory element involved in the MeJA-responsiveness
	TGACG-motif	TGACG	cis-acting regulatory element involved in the MeJA-responsiveness
Hormone response-SA	TCA-element	CCATCTTTTT	cis-acting element involved in salicylic acid responsiveness
		TCAGAAGAGG	cis-acting element involved in salicylic acid responsiveness
Hormone response-Others	O2-site	GATGA(C/T)(A/G)TG(A/G)	cis-acting regulatory element involved in zein metabolism regulation
		GATGACATGG	cis-acting regulatory element involved in zein metabolism regulation
		GATGATGTGG	cis-acting regulatory element involved in zein metabolism regulation
		GTTGACGTGA	cis-acting regulatory element involved in zein metabolism regulation
Light responsive element	3-AF1 binding site	TAAGAGAGGAA	light responsive element

	AAAC-motif	CAATCAAAACCT	light responsive element
	ACA-motif	AATCACAACCATA	part of gapA in (gapA-CMA1) involved with light responsiveness
	ACE	CTAACGTATT	cis-acting element involved in light responsiveness
	AE-box	AGAAACAA	part of a module for light response
		AGAAACTT	part of a module for light response
	Box 4	ATTAAT	part of a conserved DNA module involved in light responsiveness
	Box II	TGGTAATAA	part of a light responsive element
	chs-CMA	TTACTTAA	part of a light responsive element
		TCACTTGA	part of a light responsive element
	GA-motif	ATAGATAA	part of a light responsive element
	Gap-box	CAAATGAA(A/G)A	part of a light responsive element
	GT1-motif	GGTTAA	light responsive element
		GGTTAAT	light responsive element
		GTGTGTGAA	light responsive element
	I-box	AAGATAAGGCT	part of a light responsive element
		AGATAAGG	part of a light responsive element
		cCATATCCAAT	part of a light responsive element
		GATAAGGGT	part of a light responsive element
		gGATAAGGTG	part of a light responsive element
		GTATAAGGCC	part of a light responsive element
		TGATAATGT	part of a light responsive element
	LAMP-element	CTTTATCA	part of a light responsive element
	L-box	ATCCCACCTAC	part of a light responsive element
	MRE	AACCTAA	MYB binding site involved in light responsiveness
	Sp1	GGGCGG	light responsive element

	TCCC-motif	TCTCCCT	part of a light responsive element
	TCT-motif	TCTTAC	part of a light responsive element
	GATA-motif	AAGATAAGATT	part of a light responsive element
		AAGGATAAGG	part of a light responsive element
		GATAGGA	part of a light responsive element
		GATAGGG	part of a light responsive element
	G-box	ACACGTGT	cis-acting regulatory element involved in light responsiveness
		CACGTC	cis-acting regulatory element involved in light responsiveness
		CACGTT	cis-acting regulatory element involved in light responsiveness
		GCCACGTGGA	cis-acting regulatory element involved in light responsiveness
Stress response-others	ARE	AAACCA	cis-acting regulatory element essential for the anaerobic induction
	DRE core	GCCGAC	cis-acting element involved in defense and stress responsiveness
	ERE	ATTTCATA	cis-acting element involved in defense and stress responsiveness
		ATTTTAAA	cis-acting element involved in defense and stress responsiveness
	F-box	CTATTCTCATT	cis-acting element involved in defense and stress responsiveness
	MBSI	aaaAaaC(G/C)GTTA	MYB binding site involved in flavonoid biosynthetic genes regulation
		TTTTTACGGTTA	MYB binding site involved in flavonoid biosynthetic genes regulation
	MYB	CAACAG	MYB binding site
		CAACCA	MYB binding site
		CCGTTG	MYB binding site
		CAACGG	MYBHv1 binding site
		TAACCA	MYB binding site
		TAACTG	MYB binding site
	MYC	CAATTG	MYC binding site
		CATGTG	MYC binding site

		CATTTG	MYC binding site
		TCTCTTA	MYC binding site
	TC-rich repeats	ATTCTCTAAC	cis-acting element involved in defense and stress responsiveness
		GTTTTCTTAC	cis-acting element involved in defense and stress responsiveness
Stress response-Wound	WUN-motif	AAATTACT	wound-responsive element
		CCATTTCAA	wound-responsive element
		TAATTACTC	wound-responsive element
Stress response-Drought	MBS	CAACTG	MYB binding site involved in drought-inducibility
Stress response-Low temperature	LTR	CCGAAA	cis-acting element involved in low-temperature responsiveness

Table S3 Expression patterns of differentially expressed *Dof* genes in two cultivar

Name	D0	D1	L0	L1
StDof1	17.37486	26.9558	46.53908	38.47186
StDof3	2.733313	7.349101	4.272046	8.517256
StDof4/CDF2	151.3494	11.01259	10.00688	14.60832
StDof5	2.892272	6.553868	24.55865	23.42705
StDof6	1092.251	595.1945	723.9707	522.952
StDof7	3.910454	6.124817	7.440089	6.173066
StDof8	23.29108	5.018216	5.872741	3.164738
StDof9	1.984291	7.197514	4.348532	5.91344
StDof10	1.024236	2.654174	2.461901	5.483512
StDof11/CDF3	73.73882	29.33768	8.444475	15.61525
StDof13	55.88742	39.86603	100.429	69.09556
StDof14	15.17315	7.731721	16.39226	18.83622
StDof15/CDF5	3945.828	2239.542	3672.682	2883.541
StDof16	46.07493	79.13918	132.8207	49.56827
StDof17	2.852303	2.401997	0.312887	2.107384
StDof18	15.66935	14.18905	11.70186	12.65676
StDof19/CDF1	418.578	400.1192	97.14375	249.777
StDof20	49.02989	31.15387	68.38581	56.07596
StDof21	1.65095	1.312348	0.930326	1.429532
StDof22	80.84089	62.42663	105.9594	97.58419
StDof23	1098.788	780.5329	2666.774	1188.799
StDof24/CDF4	1945.011	252.0782	591.5836	350.323
StDof25	6.099142	0	8.916307	12.63689
StDof26	22.06998	51.34712	37.32841	34.21676
StDof27	69.65697	53.41893	58.66608	88.76452
StDof28	2.723942	1.728871	0	2.766969
StDof29	148.6724	63.0231	104.0382	92.83467
StDof30	88.72846	142.0566	132.2589	140.8398
StDof31	9.495819	15.2868	20.28894	15.10855
StDof32	44.00987	48.04436	50.28927	58.32613
StDof33	31.54218	20.44799	25.4629	28.59377
StDof34	113.573	133.4192	122.4975	162.1241
StDof35	63.15857	44.19971	16.16419	20.85008

Table S4. Regulatory network mediated by lncRNAs and their target Dofs

Dof	LncRNA
StDof36	MSTRG.229
StDof1	MSTRG.229
StDof2	MSTRG.3012
StDof2	MSTRG.3022
StDof3	MSTRG.5249
StDof3	MSTRG.5250
StDof3	MSTRG.5252
StDof4	MSTRG.5341
StDof5	MSTRG.5914
StDof5	MSTRG.5920
StDof5	MSTRG.5926
StDof8	MSTRG.6075
StDof9	MSTRG.7023
StDof9	MSTRG.7025
StDof10	MSTRG.7025
StDof10	MSTRG.7045
StDof10	MSTRG.7046
StDof11	MSTRG.7203
StDof12	MSTRG.7637
StDof12	MSTRG.7640
StDof13	MSTRG.9409
StDof13	MSTRG.9410
StDof14	MSTRG.9843
StDof14	MSTRG.9845
StDof14	MSTRG.9846
StDof14	MSTRG.9847
StDof15	MSTRG.10114
StDof16	MSTRG.10635
StDof17	MSTRG.13169
StDof18	MSTRG.13860
StDof24	MSTRG.18728
StDof24	MSTRG.18731
StDof25	MSTRG.18802
StDof25	MSTRG.18814
StDof26	MSTRG.19137
StDof26	MSTRG.19147
StDof27	MSTRG.19186
StDof28	MSTRG.22514
StDof29	MSTRG.24696
StDof30	MSTRG.24921
StDof30	MSTRG.24923
StDof30	MSTRG.24927
StDof32	MSTRG.29093
StDof33	MSTRG.30183
StDof33	MSTRG.30188
StDof33	MSTRG.30199
StDof33	MSTRG.30202
StDof34	MSTRG.31756
StDof35	MSTRG.32187
StDof35	MSTRG.32194
StDof35	MSTRG.32198
stu-miR8020	MSTRG.19137
stu-miR8045	MSTRG.18731
StDof19	StFLORE
